# Cross-calibration of a combined electrostatic and time-of-flight analyzer for energy- and charge-state-resolved spectrometry of tin laser-produced plasma

**DOI:** 10.1007/s00340-022-07767-1

**Published:** 2022-02-05

**Authors:** L. Poirier, A. Bayerle, A. Lassise, F. Torretti, R. Schupp, L. Behnke, Y. Mostafa, W. Ubachs, O. O. Versolato, R. Hoekstra

**Affiliations:** 1 Advanced Research Center for Nanolithography (ARCNL), Science Park 106, 1098 XG Amsterdam, The Netherlands; 2 Department of Physics and Astronomy and LaserLab, Vrije Universiteit Amsterdam, De Boelelaan 1081, 1081 HV Amsterdam, The Netherlands; 3 Zernike Institute for Advanced Materials, University of Groningen, Nijenborgh 4, 9747 AG Groningen, The Netherlands

## Abstract

We present the results of the calibration of a channeltron-based electrostatic analyzer operating in time-of-flight mode (ESA-ToF) using tin ions resulting from laser-produced plasma, over a wide range of charge states and energies. Specifically, the channeltron electron multiplier detection efficiency and the spectrometer resolution are calibrated, and count rate effects are characterized. With the obtained overall response function, the ESA-ToF is shown to accurately reproduce charge-integrated measurements separately and simultaneously obtained from a Faraday cup (FC), up to a constant factor the finding of which enables absolute cross-calibration of the ESA-ToF using the FC as an absolute benchmark. Absolute charge-state-resolved ion energy distributions are obtained from ns-pulse Nd:YAG-laser-produced microdroplet tin plasmas in a setting relevant for state-of-the-art extreme ultraviolet nanolithography.

## Introduction

Laser-produced plasma (LPP) has found its way to a variety of applications in science and technology ranging from laser-induced breakdown spectroscopy [1] and plasma accelerators [2], to sources of extreme-ultraviolet (EUV) light for nanolithography [3]. Sources of EUV light rely on the efficient production of highly-charged ions in the hot ($\sim 100$ eV) and dense ($\sim 10^{19-21}$ $\text{e}^-$/$\text{cm}^3$) plasma generated by pulsed laser light with intensities spanning from $10^{9}$ to $10^{11}$ W/$\text{cm}^2$ [4]. For nanolithographic tools the wavelength of choice is 13.5 nm, as for that EUV wavelength multilayer optics exist [5, 6]. For LPP based generation of 13.5 nm EUV light the species of choice is Sn, for which, due to its particular electronic structure [7], a wide range of charge states ($9+$ to $15+$) emit in a narrow band around 13.5 nm. Besides the desired EUV light, the violently expanding Sn plasma generates ionic “debris” that may affect the lifetime of the light-collecting multilayer optics by, e.g., coating, implantation or sputtering depending on the energy and charge state of the ions coming from the plasma. Measurements of the energy and charge state distributions of Sn ions coming from the LPP also give access to the expansion dynamics of the plasma itself [8–10]. Moreover, accurate data on the energy distributions facilitate the determination of the fraction of the total energy that is carried by the plasma ions [11].

A whole arsenal of techniques and instrumentation [8, 12–17] has been used to gather information on the ions coming from an LPP plasma. Ion charge-energy spectra from tin LPP have been obtained using electrostatic probes [18], Faraday cups (FCs) without [9, 10] or with retarding fields applied (RFA) [14, 15], electrostatic analyzers (ESA) [16, 17, 19, 20], or Thomson Parabolas (TP) [8]. The simplest measurement tool is the FC with which one can measure the plasma ion current. In combination with time-of-flight (ToF) measurements which become possible for pulsed operation of the LPP source, one obtains access to the energy of the ions. Placing retarding field grids in front of the FC turns the FC into an RFA, though unraveling charge state and energy is challenging. For high-resolution energy measurements, electrostatic analyzers (ESA) are optimal. However, since ESA spectrometers resolve on energy-over-charge-state (equal $E/z$ values are transmitted), charge-state-dependent energy spectra require the incorporation of ToF techniques. The absolute detection efficiency calibration of the whole system is complicated, in particular for low-energy heavy ions such as Sn ions, since detection efficiencies of channeltron electron multipliers (CEM) or microchannel plates (MCP) are very low at energies below about 2 keV and are strongly dependent on ion energy [21, 22].

In this work, we detail the absolute calibration of the ARCNL ESA operated in a combined energy and ToF mode to extract the kinetic energy distributions for the different charge states of Sn ions coming from a Sn LPP EUV source. Typically the Sn ions coming from the plasma are in charge states of $1+$ to $8+$ and have energies ranging up to several keV. After the description of the experimental setup, the following three aspects of the operation and calibration of our ESA-ToF spectrometer will be discussed in greater detail: (i) particle detection and counting procedures; (ii) the resolution of the ESA; (iii) the detection efficiency of the channeltron used for ion counting. These topics lay the basis of the energy-dependent calibration of the ESA-ToF system. Finally the independent calibration of the system will be compared to the result of a FC measurement. By means of this cross-calibration relative to the FC, the absolute calibration of the ESA-ToF analyzer is achieved. This calibration is conducted on a microdroplet tin plasma driven by a 1-$\upmu$m wavelength ns-pulsed laser under conditions relevant for producing EUV light.

## Experimental setup and methods

### Laser-produced plasma

**Fig. 1:**
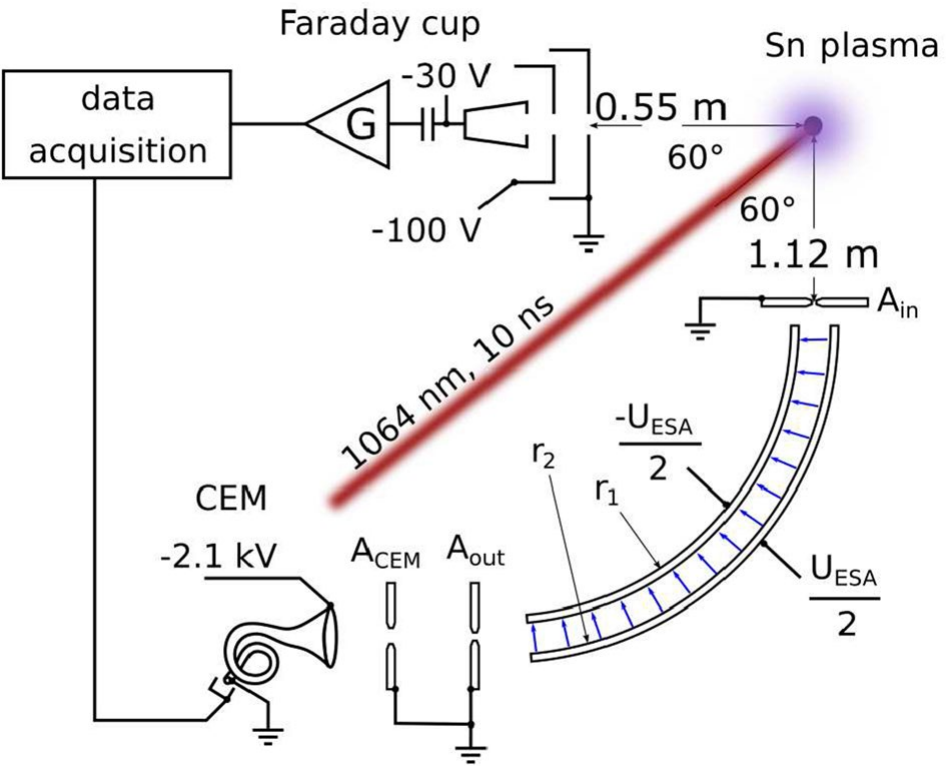
Diagnosing ion emission from Nd:YAG-produced plasma: an amplified (G), biased Faraday cup (FC) and the electrostatic analyzer (ESA-ToF) are positioned at the same angle with respect to the Nd:YAG beam direction (see main text)

Our LPP EUV source and auxiliary equipment has been described in detail before [23]. The components most relevant to this work are briefly recalled here, see also Fig. 1. A tin reservoir is mounted on top of a vacuum chamber ($10^{-7}$ mbar) and is kept at a constant temperature of $260\,^\circ$C. From the reservoir, a nozzle produces a 31.5 kHz droplet train of pure, molten tin droplets traveling along the vertical axis of the vacuum chamber. The microdroplets, which have a diameter of 28 $\upmu$m, first traverse a horizontal light sheet produced from a helium-neon laser. The light scattered off of the Sn droplets is detected by a photo-multiplier tube. The detected signal is frequency down-converted to 10 Hz to enable triggering of the laser system that creates the plasma. This Nd:YAG laser system produces pulses at 1064 nm wavelength of 10 ns time duration at full-width at half-maximum (FWHM). The beam is focused to a Gaussian spot (63 $\upmu$m FWHM) onto the droplets in the center of the chamber. Using a half-wave plate and a thin-film polarizer, the laser pulse energy can be adjusted without affecting the spatial beam profile. In the following, the laser pulse energy was set to 112 mJ unless otherwise specified.

### The ESA-ToF energy analyzer

After a 1.12-m flight path from the LPP, ions originating from the plasma enter the ESA through an input aperture $\mathrm{A_{in}}$ with a diameter of about $60\,\upmu$m. The ESA is positioned at an angle of 60$^{\circ }$ with respect to the incoming laser beam, in mirror symmetry with a FC. The radii of the inner and outer deflection electrodes of the ESA are respectively $r_1$ = 190 mm and $r_2$ = 210 mm. Symmetrical bias voltages of $\pm U_{\mathrm{ESA}}/2$ generate a radial electric field which forces positively charged ions of a specific energy-over-charge ratio onto a circular trajectory in between the cylindrical electrodes (85$^\circ$ in arclength). Thereafter selected ions pass through two grounded apertures $\mathrm{A_{out}}$ (inner diameter 2.5 mm) and $\mathrm{A_{CEM}}$ (inner diameter 3.4 mm), which are 5.5 mm apart. Because $\mathrm{A_{in}}$ is much smaller than $\mathrm{A_{out}}$, the geometrical transmission of the ESA is close to unity as long as space-charge-driven beam expansion is not significant. The output apertures shield the electric fields from the ESA plates from the field of the channeltron electron multiplier detector and vice versa. This allows for changing the voltages on the CEM without affecting the ESA, which is important to determine the detection efficiency of the CEM for Sn ions of different energy and charge state (see below).

The pass energy $E_0$, the energy at which ions in charge state $z$ can traverse the ESA without being intercepted, is related to the voltage difference between the electrodes $U_{\mathrm{ESA}}$ via [24]

1 $$\begin{aligned} \frac{E_0}{z} = \frac{e\,U_{\mathrm{ESA}}}{2 \ln (r_2/r_1)}, \end{aligned}$$

where *e* is the elementary charge. For our ESA, $E_0/z$ [in eV] is $5.0 \times U_{\mathrm{ESA}}$, with $U_{\mathrm{ESA}}$ in V. The ESA filters ions by their energy-to-charge ratio, consequently 1 keV $\text{Sn}^{1+}$ ions cannot be distinguished from 2 keV $\text{Sn}^{2+}$ ions, 3 keV $\text{Sn}^{3+}$ ions, and so on. For ions with the same energy-over-charge ratio, the higher charged ions have higher energy and thus have a shorter ToF from LPP to ESA. Therefore, by measuring the ToF of the ions passing through the ESA, one can resolve the ions of different charge states. A 600-MHz Keysight Infiniium oscilloscope with an input impedance of 50 $\Upomega$ is used to record the time of their individual arrival.

**Fig. 2:**
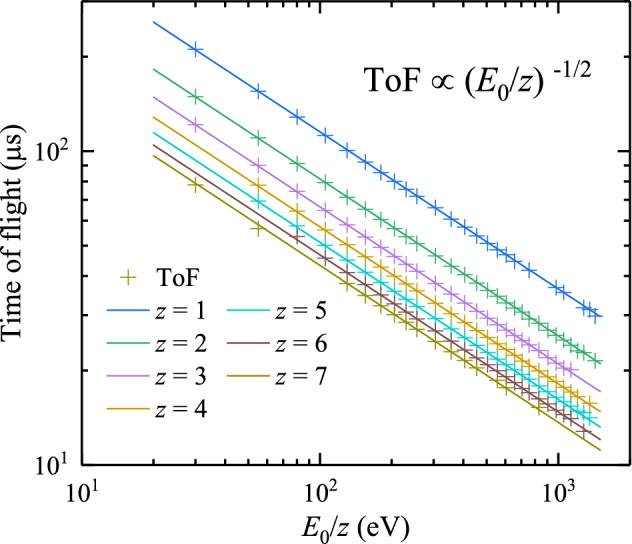
Measured average time of flight of $\text{Sn}^{z+}$ ions in charge states of $z=1{-}7$ (colored crosses) for 30 values of the *z*-scaled pass energy, $E_{0}/z$. The lines represent fits to the data with the flight path $L$ as a common fit parameter. The fit yields an ion path $L=1.470(2)$ m

**Table 1:** Masses and natural abundances of stable Sn isotopes

Isotope	Mass (amu)	Abundance ($\times 10^{-3}$)
112	111.9	9.7
114	113.9	6.6
115	114.9	3.4
116	115.9	145.4
117	116.9	76.8
118	117.9	242.2
119	118.9	85.9
120	119.9	325.8
122	121.9	46.3
124	123.9	57.9

To obtain a charge-state-resolved ion energy spectrum, the pass energy $E_{\mathrm{0}}$ of the ESA is scanned over the interval of interest by changing the voltage $U_{\mathrm{ESA}}$. For each $U_{\mathrm{ESA}}$ value, ToF detector traces are typically acquired for 200 laser shots. Each ToF trace shows distinct current transients grouped in several ToF intervals. The center-of-mass of such a charge state cluster arrives at a ToF given by $t=L\sqrt{M/2 E_0}$, with $L$ being the total distance between LPP and the CEM detector and $M$ the average mass of the Sn particles. Sn has 10 stable isotopes with natural abundances of a couple of percent or more. The most abundant isotopes are $^{118}$Sn (24.2%) and $^{120}$Sn (32.6%), see Table 1. The average mass of Sn based on its natural abundances is 118.71 amu.

Using this average mass of Sn, Fig. 2 shows the ToF of ion bunches for different pass energies and charge states. In the figure, the lines are $L\sqrt{M/2 E_0}$ fits, with $L$ as a global fit parameter. The fits yield a common $L$ value of 1.470(2) m, which is in close agreement with the actual measured distance of 1.476 m. The fact that for all energies and charge states the data are almost perfectly fitted indicates that effects of stray electric and magnetic fields can be neglected, since they would affect different charges and energies differently.

The charge state resolution is limited by the width of the isotope distribution. For $\text{Sn}^{z+}$ with the same pass energy [Eq. (1)], at a specific charge state $z_{\mathrm{max}}$ the lightest isotopes of that charge state have the same ToF as the heaviest isotopes with charge $z_{\mathrm{max}}+1$. The isotope distributions of adjacent charge states start to overlap. For Sn ions this happens in our analyzer at $9+$.

The individual ToF traces are post-processed via software for an optimal registration of particle counts. Corrections for count rate or dead time effects can subsequently be applied (see below). After sectioning the ToF traces into different charge state clusters, an algorithm looks for individual transients with amplitudes larger than a given threshold, separated by a minimal time $\tau$ set by the dead time of the channeltron. In case of high count rates, a correction factor for pile-up effects $\eta _{\mathrm{CR}}$ is determined by considering the yields of the Sn isotopes separately, see Sect. 3.1.

After the count rate corrections, two more instrumental correction factors need to be considered for the conversion of the numbers of detected ions per each charge per pass energy into a charge-state-dependent energy distribution, $dN_z(E)/dE$. Namely, those corrections are the pass-energy-dependent energy-bin width $\Delta E \left( E \right)$, and the energy- and charge-state-dependent detection efficiency $\eta _{\mathrm{det}}$.

Our type of ESA operates in a fixed $\Delta E \left( E \right) /E$ mode [24], implying that the energy interval within which ions can pass the ESA increases linearly with pass energy. Based on its mechanical properties one expects a $\Delta E\left( E \right) /E$ ratio close to 1% following Ref. [24].

In Sect. 3.3 the energy- and charge-state-dependent detection efficiency $\eta _{\mathrm{det}}$ of the channeltron (a gridded Photonis 4502 extended dynamic range channeltron) mounted to the ESA-ToF system is determined. The impact energy ($E_{\mathrm{imp}}$) of the Sn ions impinging on the channeltron determines the detection efficiency $\eta _{\mathrm{det}}$ and is equal to $(E_0 + zU_{\mathrm{head}})$ with $E_0$ the pass energy of a $\text{Sn}^{z+}$ ion and $U_{\mathrm{head}}$ the voltage on the head of the channeltron. By scanning the voltage on the head of the channeltron while keeping the pass energy fixed, the energy dependence of $\eta _{\mathrm{det}}$ is established. For a complete mapping of the dependence of the detection efficiency on charge state and energy, the procedure is repeated for a series of pass energies and charge states.

Finally, to assess the overall calibration of our ESA-ToF analyzer, a cross-calibration with Faraday cup data is conducted. This requires the solid angle $\Delta \Omega$ of the ESA-ToF to also be taken into account. The comparison with the charge-integrated FC data is carried out by summing the contributions from different charge-state-dependent ion energy distributions ${\mathrm{d}}^2N_z(E)/{\mathrm{d}}E{\mathrm{d}}\Omega$:

2 $$\begin{aligned} \frac{{\mathrm{d}}^2 Q}{{\mathrm{d}}E{\mathrm{d}}\Omega } = \sum _z e z \frac{{\mathrm{d}}^2 N_z}{{\mathrm{d}}E{\mathrm{d}}\Omega } \approx \sum _z \frac{e z N_z^{\mathrm{m}}(E)}{\eta _{\mathrm{CR}}\,\eta _{\mathrm{det}}\,\Delta E\left( E \right) \,\Delta \Omega }, \end{aligned}$$

with the last equality true up to a common prefactor of order unity value, related to the definition of the resolving power $\Delta E$ (see Sect. 3.2). $N_z^{\mathrm{m}}(E)$, the single-shot measured number of ions with energy in the interval $E\pm (\Delta E (E)/2)$, is corrected by $\eta _{\mathrm{CR}}$ and $\eta _{\mathrm{det}}$. Converting these distributions [Eq. (2)] into an integral ion current enables a straightforward comparison to the FC measurements.

### Faraday cup

At an angle of 60$^{\circ }$ with respect to the incoming laser beam (see Fig. 1), a Faraday cup is mounted onto the LPP chamber to ensure comparability with the ESA-ToF measurements. The FC (FC-73 of Kimball Physics) with an entrance aperture diameter of 5 mm collects charge-integrated ion current at a distance of 55 cm from the LPP. The FC consists of a grounded shield, a suppressor grid negatively biased at $-100$ V, and a collector cup biased at $-30$ V (refer to the sketch of Fig. 1). The negative bias on the suppressor grid and cup blocks electrons emanating from the plasma, and suppresses the escape of secondary electrons from the cup.

**Fig. 3:**
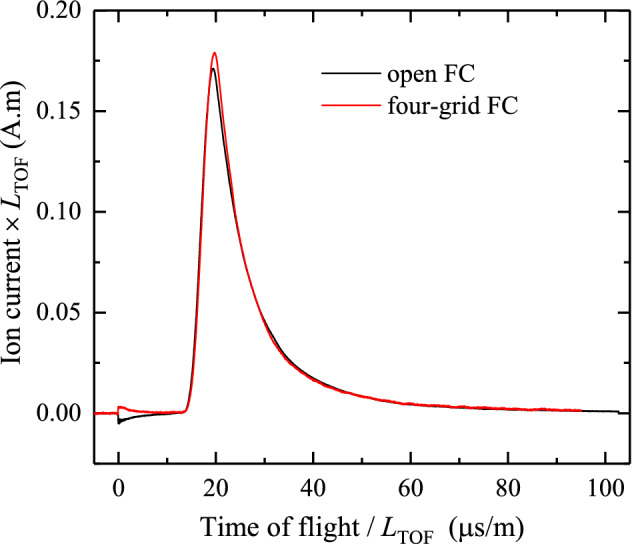
Examples of ion currents as measured by the grid-less, open FC (black line) and by the four-grid FC (red line) corrected for a transmission of 41%. Signals were not amplified. The time of flight and the ion current axes are corrected for the slight difference in distance to the plasma (here 0.62 m for the open FC and 0.67 m for the gridded FC)

For its potential use as a Retarding Field Analyzer, the FC contains four grids, each with a given optical transmission of 80% per grid reducing the overall transmission to 41%. The signal measured by the FC-73 was compared to that measured by an open (grid-less), in-house built FC, placed at the same angle with respect to the laser direction. Figure 3 shows the two ToF traces, corrected to account for small differences in distance to the plasma and for detection solid angle. For both FCs, the signal was not amplified but instead measured across a 10 k$\Upomega$ shunt resistor. The two measured ion currents are in very good agreement. The remaining small difference at $t=0$ ns is indicative of the fact that the gridded FC provides for better suppression. In the subsequent comparison with electrostatic analyzer data, the signal of the gridded FC is used, corrected for the here validated $41\%$ transmission factor.

**Fig. 4:**
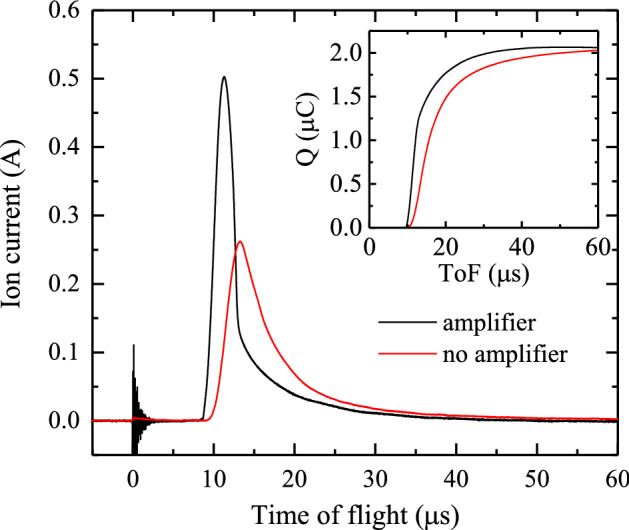
The amplified signal of the gridded FC (black line) vs raw signal (red line). The total collected charge (inset) is very similar for the two readout methods, with an accumulated charge of 2.0 $\upmu$C some 60 $\upmu$s after the generation of plasma

In the following measurements, with the aim to facilitate a comparison of FC and ESA-ToF, we buffer the gridded FC with a trans-impedance amplifier with a gain of 25 kV/A and a bandwidth of 25 MHz. The amplifier consists of two stages. The first, a trans-impedance stage with 5 kV/A gain, is based on a high speed operational amplifier (OPA847 of Texas Instruments) with a low input current noise of 2.5 pA/$\sqrt{\text{Hz}}$ and a gain bandwidth product of 3 GHz. This stage is followed by a non-inverting output stage (OPA691) with tenfold amplification. The output impedance of the amplifier is 50 $\Upomega$. Due to the fast response of the amplifier, deconvolution of the measured signal from the measurement electronics is not necessary to accurately reflect the ion current impinging upon the collector.

An additional experiment was conducted to confirm the gain of the trans-impedance amplifier. Using a stable tin droplet plasma source for generating ions, the ion current was assessed twice with the same FC: with and without amplification. The ToF traces are presented in Fig. 4. The fast response time of the amplifier is apparent in the amplified signal (black line), whereas the raw signal (red line) suffers from a large RC time, on the order of several microseconds (consistent with an expected RC time of $\mathrm{RC} \approx 10\,\mathrm{k}\Upomega \times 400\,\text{pF} = 4.0\,\upmu$s). Both time-integrated currents (i.e., the time-dependent collected charge) converge to a common value of $\sim 2.0\,\upmu$C, as illustrated in the inset. The amplifier gain is therefore established to be equal to its expected value of 25 kV/A. Measured ion transients have a typical duration on the order of several 10 $\upmu$s. Our amplifier, providing an amplified capacitive readout of the FC, was designed accordingly to have a fast current ramp-up (ns) and a particularly slow (negative) charge re-flow (tens of ms).

## Characterization of the ESA-ToF energy analyzer

### Count rate effects

Averaged over the full duration of a ToF spectrum taken at a specific scaled pass energy ($E_0/z$), the count rates are not very high, but as shown in Sect. 2.2 the ions arrive at the detector in single charge state batches. Typical bunch lengths are on the order of 1 $\upmu$s, therefore a batch of just 100 $\text{Sn}^{z+}$ ions corresponds to a rate of no less than 100 MHz. Even for instrumental dead times of the order of nanoseconds, such high count rates imply that the different types of detector dead time need to be considered.

**Fig. 5:**
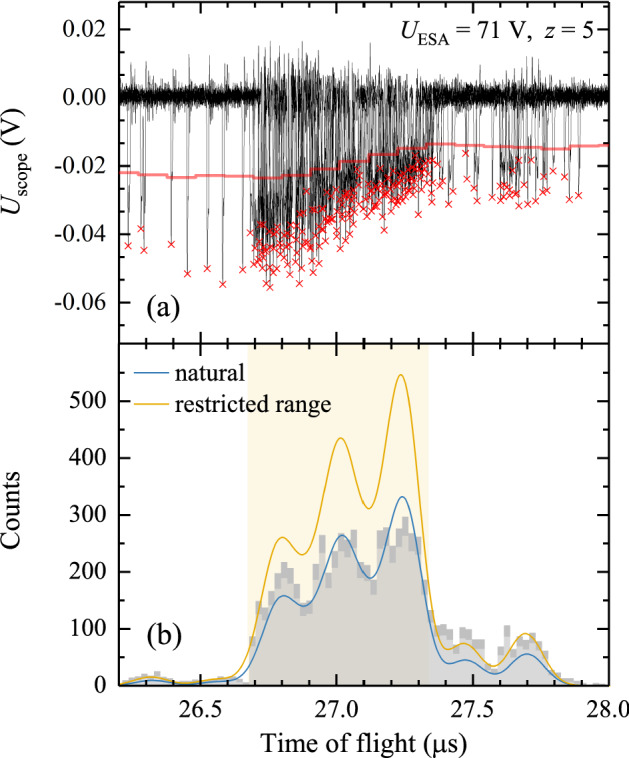
Panel **a** shows examples of ESA-ToF transients built up out of five traces of 1775 eV $\text{Sn}^{5+}$ (scaled pass energy $E_0/z$ = 355 eV). For all traces the ions stem from a LPP plasma produced by 10-ns, 60 mJ laser pulses. The red line indicates the threshold level used to discriminate counts, which are represented by the red crosses. **b** The corresponding ToF histogram (200 laser shots included) is fitted assuming a natural Sn isotope distribution (blue line). A similar fit (yellow line) is performed while using only the low-abundance, lightest and heaviest isotopes (ranges outside the shaded yellow area), which have the lowest count rate and are the least likely to be affected by high count rates. The statistical error in the number counts per bin is shown in dark gray

When a single Sn ion impinges onto the head of the channeltron and releases an electron from the channeltron’s surface, it triggers an avalanche of electrons, which is sustained by a large bias voltage (typically in the 2–3 kV range) across the channeltron. The avalanche of electrons is collected by an anode at the tail of the channeltron, producing a current transient. This transient charge current associated with the avalanche of electrons is a few ns long and can be picked up by standard data acquisition electronics (we use a 600 MHz Keysight Infiniium oscilloscope). Such an electron avalanche temporally lowers the potential over the channeltron. Subsequent ion impacts on the channeltron may thus produce signals with a smaller amplitude until the gain of the detector is fully recovered. This phenomenon is exemplified in Fig. 5.a where a height distribution of channeltron pulses is shown for $\text{Sn}^{5+}$ ions at a scaled pass energy $E_0/z$ of 355 eV which corresponds to a kinetic energy of 1775 eV for $\text{Sn}^{5+}$ ions. Time of flight traces accumulated over five laser-shot produced LPP plasmas are shown. As expected, a decrease in the signal amplitude is observed at high count rates. While at the start of the batch a $-25$ mV detection threshold would be appropriate to distinguish real counts from background and noise, later on in the batch such a threshold would rule out some real counts. By making use of a dynamic threshold equal to half of the local average amplitude of the signal peaks, an unnecessary loss of counts is prevented. The ion detection peak signals are several ns wide, and often extended with a shoulder feature and low-amplitude ringing signals. By setting a 10 ns-wide software dead time, it is ensured that these features are not counted separately. This software dead time of 10 ns is taken as the (non-extending) dead time $\tau _{np}$ of the ESA-ToF detector.

Depending on the properties of the detector system, two basic types of dead time can be identified, e.g. [25], (i) non-extending (or non-paralyzed) or (ii) extending (or paralyzed) ones. Basically, after the registration of a first particle on the detector, if a second one hits the detector within the dead time ($\tau$) of the system, depending on the detector properties, the system’s dead time will not (type i) or will (type ii) be extended. Being able to judge what is the leading type of deadtime requires an accurate knowledge of the types and numerical values of all relevant dead times. In a wide variety of fields, from nuclear and high-energy detector research, via photon science at synchrotrons, to biomedical imaging e.g. [26–29] further extensions are made, for instance by introducing longer software dead times, determining time distributions between recorded events, or using hybrid dual dead time descriptions to cope with high count rates.

Given the 10-ns software deadtime, which is relatively long compared to the transient pulse width, we can effectively view our detector as having a constant, non-extending dead time. For a detector with such a constant dead time, $\tau _{np}$, to first order the measured count rate ($R_m$) connects to the actual incoming particle rate on the detector ($R$) as

3 $$\begin{aligned} R_m = \frac{R}{1+R\tau _{np}}. \end{aligned}$$

**Fig. 6:**
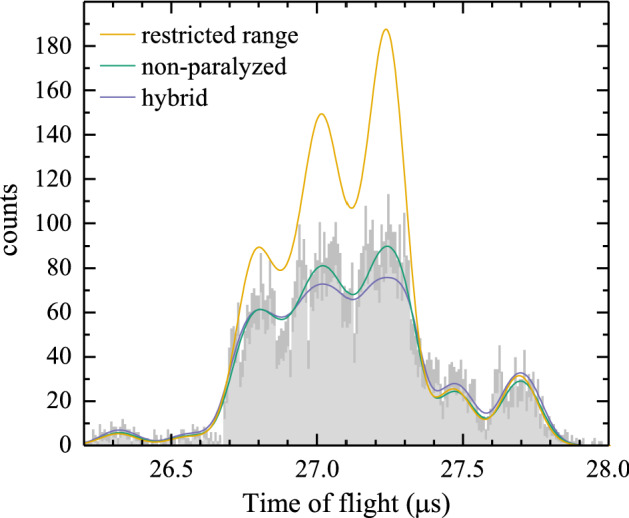
ToF histogram of $\text{Sn}^{5+}$ ions of 1775 eV. Fits of the non-paralyzed (green line) and the hybrid (purple line) models to the data are shown. The range-restricted model from Fig. 5 is also presented. Statistical errors of the bin heights are shown in dark gray. The data are identical to those shown in Fig. 5 but with thrice the number of bins (see main text). The detected number of ions in the histogram $N_z^{\mathrm{m}}(E)$ is 7375, and the real number of ions $N$ as recovered from the model fits is 12603 and 15455 for the non-paralyzed and hybrid models respectively

For the ESA-ToF system used to diagnose Sn ions coming from the Sn droplet LPP, the natural isotope distribution of Sn enables us to assess the validity of count rate corrections based on Eq. (3).

Figure 5b illustrates the need for isotope-specific count rate corrections. The figure presents a histogram of detected ion transients resulting from the acquisition from 200 laser shots on droplets (the results of five acquisitions are shown in Fig. 5a for clarity). The blue line is a least-square fit to the histogram of a sum of Gaussian functions with equal width, and with amplitudes following the natural isotope abundance of Sn. The three free fit parameters are (i) Gaussian width $\sigma$, (ii) amplitude and (iii) centroid time of flight position.

The fit underestimates the amplitudes corresponding to the heavier and the lighter isotopes. These count rate effects may be highlighted by restricting the fitting range of the histogram to those sections of the histograms where ion counting is not dead-time-limited, i.e. the low-abundance isotopes. The yellow line represents the result of such a fit that excludes the area (shaded yellow in Fig. 5b) where count rate effects will be most pronounced. The result clearly demonstrates the under-counting of in particular the most abundant isotopes. The true number of impinging ions could be obtained from the integral of such range-restricted fitting. This approach would however eliminate most of the counts from the fitting procedure, thereby deteriorating the statistical confidence in the fit. Therefore, in the following, we employ Eq. (3) to obtain the true count rates using all detected hits.

In Fig. 6, the ToF histogram of the measured number of ions ($S_m$) for $U_{\mathrm{ESA}}=71$ V and $z=5$ is fitted with the non-paralyzed model (green line) using Eq. (3) in tandem with a spectral distribution function based on the natural isotope abundances ($F_i$, see Table 1) of Sn and Gaussian ToF distributions $G_i(t,t_i,\sigma )$ for each of the isotopes, with $t$ the time of flight, $t_i$ the centroid ToF of isotope $i$, and $\sigma$ the preset Gaussian width common to all isotopes (see below Sect. 3.2). The spectral distribution function is thus defined as $\sum _i F_i G_i(t,t_i,\sigma )$. This yields the following description of the measured number of counts:

4 $$\begin{aligned} S_{\mathrm{m}}(t)=R_mT_m=\frac{N \sum _i F_i G_i(t,t_i,\sigma )}{1+\sum _i F_i G_i(t,t_i,\sigma )N\tau _{\mathrm{np}}/T_{\mathrm{m}}}. \end{aligned}$$

$T_m$ is the time over which the counts per bin are accumulated and is equal to number of acquisitions $N_{\mathrm{tr}}$ times the bin width $\Delta t$. A common way to estimate the number of bins needed to sample a Gaussian function is the square root of the number of counts. Here we deal with 10 isotopes with partly overlapping peaks, therefore the number of bins is taken as three times the square root of the number of hits. The histogram bins are distributed over a time window defined by the time of flight of virtual tin isotopes with 1 mass unit less than the lightest stable isotope and 1 mass unit heavier than the heaviest one. Note that the sum of $S_{\mathrm{m}}(t)$ over the bins within the time window equals the total measured number of ions in charge state $z$ evaluated at kinetic energy *E*, $N_z^{\mathrm{m}}(E)$. In addition it should be realized that the width of the time window scales inversely with the ToF of the ions. The higher the energy of the particles, the narrower the time window.

The fit parameter of prime interest is $N$, the total number of ions triggering the detector. For comparison purposes, the range-restricted fit (yellow line) is shown in Fig. 6 along with a hybrid count rate model fit [30] (purple line), in which an additional paralyzed dead time of 1.5 ns is included. A good agreement between the histogram and the non-paralyzed and hybrid models is observed and the heights of all peaks are properly reproduced, in particular by the non-paralyzed model. Even for this maximum count rate case, a relatively small $\sim$20% difference in true number of hits $(N)$ was obtained comparing the non-paralyzed and hybrid models (for most data this difference is much lower, at the percentage level, see below). Therefore in the following the simpler non-paralyzed model is used. The ratio between $N_z^{\mathrm{m}}(E)$ and $N$ is the correction factor $\eta _{\mathrm{CR}}$ introduced in Sect. 2.2. The model-related uncertainty can be gauged by comparing the non-paralyzed and range-restricted fits, which differ 7% on average, a number that can be considered as being representative for the model uncertainty. The average difference between hybrid and non-paralyzed outcomes is a similar 6%.

**Fig. 7:**
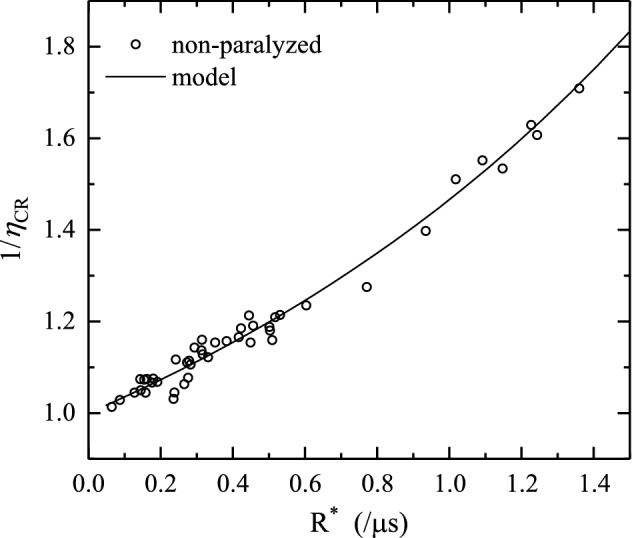
The inverse count rate correction factor $1/\eta _{\mathrm{CR}}$ versus scaled count rate, $R^*$. The data are obtained by fitting Eq. (4) to the measured spectra. The analytical trend (black line) is calculated on basis of Eq. (4), using a width of the Gaussian functions corresponding to an energy resolution of $\alpha _E$ of $1.1\%$ (see Sect. 3.2)

Figure 7 summarizes the count rate correction factor results obtained from fitting Eq. (4) to a large set of ToF spectra covering many charge states, energies (which correspond to different ToF ranges) and count rates. The inverse count rate correction factor $1/\eta _{\mathrm{CR}}$ is shown as a function of a scaled effective count rate, which allows for the use of a single set of $\eta _{\mathrm{CR}}$ values for high count rate correction independent of the ToF range in which the ions are detected. Here, the average scaled count rate $R^*$ is defined as $N_z^{\mathrm{m}}(E)/\mathrm{ToF}_z(E)$, with $\mathrm{ToF}_z(E)$ the average ToF of all Sn isotopes in charge state $z$ and with energy $E$, which corresponds to the ToF of a Sn particle of average mass of 118.7 amu. $R^*$ takes out the fact that the width of the ToF window in which all isotopes of a specific charge state $z$ and energy $E$ arrive at the detector scales linearly with the ToF. Given that the number of sampling bins is taken proportional to the square root of the number of detected ions $N_z^{\mathrm{m}}(E)$, for the same number of detected ions, the sampling bin widths and thus the count rate scale with $1/\mathrm{ToF}_z(E)$. The excellent agreement between model and data shows that with $R^*$ as parameter, a common set of correction factor $\eta _{\mathrm{CR}}$ can be used to correct the detected number of ions for possible high count rate effects. Another benefit is that one does not need to fit every spectrum to obtain the true number of ions; it suffices to count the number of detected hits in the validated range (cf. Fig. 7) of count rate values and the corresponding pass energies.

### Energy resolution

As explained in Sect. 2.2, the energy resolution of the ESA-ToF is required to derive absolute ion energy distributions [cf. Eq. (2)]. A cylindrical ESA has a constant $\alpha _E\equiv \Delta E/E$ energy resolution. Experimental data feature smooth Gaussian-like distributions around each isotopic peak, whereas an ideal ESA would present a discrete and sharp “block” acceptance interval determined by the geometry of the ESA. The absolute resolution $\Delta E$ is chosen to be equal to $w_E$, the FWHM of the Gaussian distributions following [24]. The time domain resolution is translated to the energy domain using $\Delta E \left( E \right) / E = 2 \Delta t \left( t \right) / t$. A common value for $\alpha _E$ was seen to be in good agreement with all data, in line with expectations. An average relative energy resolution $\alpha _E \equiv w_E/E_0 = 1.1\,\%$ was extracted by fitting all ToF histograms.

### Detection efficiency

The detection efficiency of a CEM depends on the probability for the impinging particles to produce secondary electrons by their impact on the CEM surface. For ions in low charge states the generation of secondary electrons is determined by kinetic electron emission, i.e., electrons are produced by the interaction of the projectile particle with electrons on the CEM surface. The average yield of secondary electrons stemming from kinetic electron emission increases with impact velocity [31, 32].

Typically, ions that carry more potential energy than twice the work function of the target material can also generate secondary electrons by means of potential-energy-driven Auger electron emission [33]. The yield of secondary electrons produced by potential energy emission is only weakly dependent on impact energy [34].

To accurately reconstruct the number of ions of different energies and charge states passing through the ESA-ToF, a careful calibration of the channeltron detection efficiency is required. Such a calibration can be performed by post-accelerating the ions that passed through the ESA by applying a suitable bias potential to the detector head. The bias voltage at the head of the CEM is stepped from zero to $-3.5$ kV in steps of $-100$ V while keeping the voltage across the CEM, and thus the gain, constant at 2145 V. For each CEM head voltage setting we scan $U_{\mathrm{ESA}}$ through a small array of voltages (12 for this calibration) to obtain a range of ion $E_0/z$ ratios. The total kinetic energy of the impinging ions is $E_0+z U_{\mathrm{head}}$, with $U_{\mathrm{head}}$ the voltage on the head of the channeltron. To obtain sufficient data for the low-energy, low-charge state ions, a hydrogen buffer gas was added (in between the LPP and the ESA-ToF) in part of the measurements. The addition of this tenuous buffer gas increased significantly the number of singly and doubly charged Sn ions as Sn ions in higher charge states are driven into lower charge states by means of electron capture from the $\text{H}_2$ gas.

**Fig. 8:**
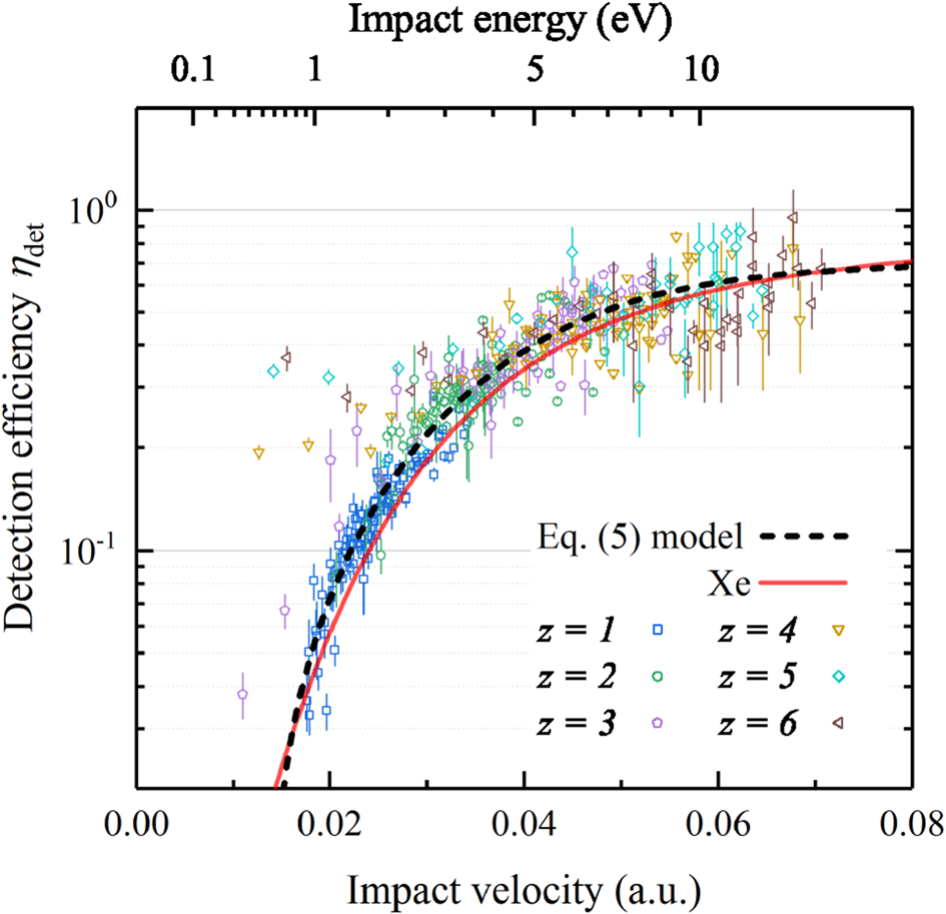
Absolute CEM detection efficiency of tin ions as function of impact velocity and energy $E+z U_{\mathrm{head}}$. The fit of Eq. (5) to the data (see main text) is shown as a black dashed line. The red line presents data from Krems et al. [22] for the detection of Xe ions

Figure 8 shows the relative detection efficiency of the ESA-ToF channeltron obtained from our calibration measurements. For all charge states of the Sn ions a monotonically increasing detection efficiency with impact velocity (deduced from the total kinetic energy) is observed. The data obtained for each charge state have an arbitrary relative scale, given that the incoming number of Sn ions of a particular charge state and energy is not known. The individual, smooth data curves can however be overlapped by minimizing the respective differences in the common impact velocity ranges. A smooth generic curve is obtained, which describes the energy-dependent calibration of the detection efficiency after setting a $\eta _\infty =70\%$ (following Krems et al. [22]) asymptotic value for the maximally obtainable detection efficiency. The thus obtained data can be compared to the measurements (red line) by Krems et al. [22] using Xe ions, being of very similar mass. There is a good agreement between their and our data. It is of note that the Xe ions have a higher potential energy which may lead to a small additional contribution to the detection efficiency of Xe ions.

For $\text{C}_{{60}}$ ions where secondary electron emission is solely induced by kinetic energy, Schlathölter *et al.* [35] used a semi-empirical description of the detection efficiency curve, which incorporates the velocity dependence of kinetic, secondary electron emission [31]:

5 $$\begin{aligned} \eta _{\mathrm{det}}(v) = a_1 \left( a_2 + \tanh \left( \frac{v - v_0}{a_3} \right) \right) , \end{aligned}$$

where $a_1 \left( a_2 + 1 \right) =\eta _\infty = 70\%$ is the maximum detection efficiency and *v* the impact velocity. According to the ion potential energy considerations raised above, only $z=1$, $z=2$ and the high-energy $z=3$ data were used for fitting. The resulting calibration curve (black dashed line) is shown in Fig. 8, and is in very good agreement with the Krems curve. The resulting fit parameters are $a_1=0.41$, $a_2=0.70$, $a_3=0.024$ a.u., $v_0=0.034$ a.u. Sn ions with higher charge states $z>2$ have higher detection efficiencies due to their additional potential energies; this effect is clearly observed at impact energies below 2 keV.

In the following, the CEM bias is set to -2050 V at which point potential emission of the higher charge states are negligible compared to the respective kinetic emission and all charge states can be well described by the single calibration curve shown in Fig. 8.

The calibrated ESA-ToF can now be employed to accurately characterize ion energy distributions from the laser-driven plasma. In the following an ESA-ToF spectrum is presented and critically compared with the charge-integrated FC trace obtained simultaneously.

## ESA-ToF and FC cross-calibration

**Fig. 9:**
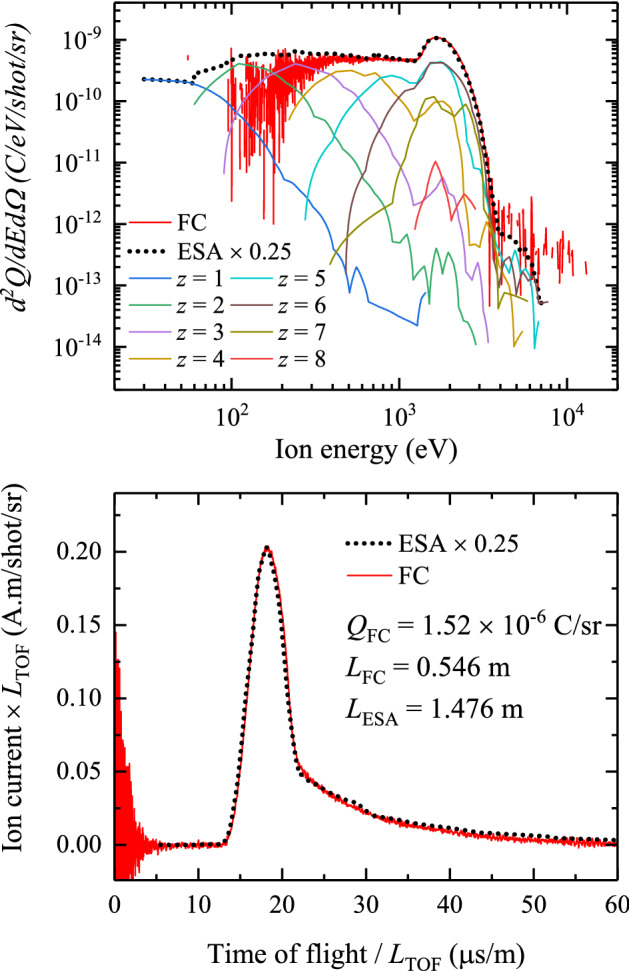
**a** Charge-state-resolved ion energy distribution of a Nd:YAG-driven tin droplet plasma for a laser energy of 60 mJ and a pulse duration FWHM of 10 ns. The charge-state-integrated energy distribution (black dotted line) as measured by the ESA-ToF is shown along with the measurement from a Faraday cup (red line). **b** Reconstructed ESA-ToF ion current transient (black dotted line) and the ion current from the Faraday cup (red line). In panels **a** and **b**, the ESA-ToF data are multiplied by an overall scale factor of 0.25 (see main text)

Figure 9 presents the absolute ion charge energy distribution and current as measured by the ESA-ToF and a FC (see Fig. 1), after applying the corrections outlined previously. The data were obtained under experimental conditions similar to those of the calibration but at a different laser pulse energy of 60 mJ.

Following Deuzeman et al. [9], the conversion from a charge current in time- to energy-domain is performed using

6 $$\begin{aligned} \frac{{\mathrm{d}}Q}{{\mathrm{d}}E} = \frac{{\mathrm{d}}Q}{{\mathrm{d}}t} \frac{{\mathrm{d}}t}{{\mathrm{d}}E} = I \frac{t}{2E}, \end{aligned}$$

where ${\mathrm{d}}Q/{\mathrm{d}}E$ and ${\mathrm{d}}Q/{\mathrm{d}}t = I$ are the signals in the energy domain (cf. Fig. 9a) and the time domain (cf. Fig. 9b), respectively. The contributions from the individual charge states measured with the ESA-ToF (colored lines, see legend) are multiplied with their respective charge $z$ to portray the energy distribution of the collected electric charge:

7 $$\begin{aligned} \frac{{\mathrm{d}}^2 Q_{z}}{{\mathrm{d}}E{\mathrm{d}}\Omega } = e z \frac{{\mathrm{d}}^2N_{z}}{{\mathrm{d}}E{\mathrm{d}}\Omega }. \end{aligned}$$

The data indicate the presence of a large range of ion charge states, up to $z=8$. Higher charge states may in fact be present in exponentially low quantities, but here cannot be reliably detected given their rarity and the maximum resolvable charge state of the ESA-ToF (see Sect. 2). The charge-state-resolved energy distributions have similar, peaked shapes. The positions of the peaks appear to be a smooth function of charge $z$. Between 2 and 3 kV the charge-state-resolved spectra exhibit a common peak, which in turn produces a peak in the charge-integrated distribution. The peak is followed by a sharp drop-off; no ions are detected above 5–6 keV.

The overall energy distribution (black dotted line) is the sum of contributions from the individual charge states:

8 $$\begin{aligned} \frac{{\mathrm{d}}^2Q}{{\mathrm{d}}E {\mathrm{d}}\Omega } = \sum _z \frac{{\mathrm{d}}^2 Q_{z}}{{\mathrm{d}}E {\mathrm{d}}\Omega } = \sum _z e z \frac{{\mathrm{d}}^2 N_{z}}{{\mathrm{d}}E {\mathrm{d}}\Omega }. \end{aligned}$$

It can be compared to the charge-integrated data acquired with the FC. Figure 9b shows the ion current detected by both instruments, and processed as described above. To facilitate a direct comparison of the measured ion currents, the time axis has been stretched by a factor $1/L_{\mathrm{ESA}}$ and $1/L_{\mathrm{FC}}$, and the current axis was multiplied by $L_{\mathrm{ESA}}$ and $L_{\mathrm{FC}}$ respectively for the FC and for the ESA-ToF. All data were corrected for solid angle. Ion current traces of 1000 laser shots were averaged for the FC and 200 laser shots for the ESA-ToF. The resulting current is corrected for the 41% transmission of the four grids. Using Eq. (6), the averaged time of flight trace (red line in Fig. 9b) is converted to the energy distribution of the total charge collected by the FC (red in Fig. 9a). Lastly, using the FC as an absolute benchmark, an overall scale factor of 0.25 was applied to the ESA-ToF’s $d^2Q/dEd\Omega$ data and corresponding ion current amplitude for optimum agreement between FC and ESA-ToF. This scale factor is related to the fact that Eq. (2) gives the total count rate up to a common pre-factor associated with the interpretation of the energy resolution $\Delta E$. We attribute the difference factor to the fact that the ESA does not exhibit the ideal rectangular pass band [24] and the practical assumption that the transmission function (resolution) is of Gaussian shape width, following Granneman [24], its full-width-half-maximum proportional to the true resolution by an instrument-specific multiplication factor. It is the relative calibration to the FC that enables obtaining this missing factor. The overall $\sim$20% calibration uncertainty is dominated by the uncertainty in establishing the resolution ($\sim 10$%), in the count rate model ($\sim$7%), and the asymptotic value of the CEM calibration curve ($\sim$10%). The now fully corrected ESA-ToF data are in excellent agreement with the FC data both in energy and ToF representation.

## Conclusion

We present a charge- and energy-resolved spectrum of Sn ions produced by laser-driven microdroplet-tin plasma relevant for the production of extreme ultraviolet (EUV) light. For this purpose, we calibrated an electrostatic analyzer used in time-of-flight mode (ESA-ToF). The calibration procedure included the characterization of the channeltron detection efficiency, the energy resolution, and the influence of (high) count rate effects - facilitated by the wide isotope distribution of tin. Charge-summed distributions obtained from the calibrated ESA-ToF are shown, after multiplication with a global constant factor, to be in excellent agreement with Faraday cup measurements (using two distinct types of FCs, with and without amplifier circuitry) further validating the calibration procedure, enabling absolute measurements of charge-state-resolved spectra to characterize and optimize future sources of EUV light.
